# Experiences in conducting multiple community-based HIV prevention trials among women in KwaZulu-Natal, South Africa

**DOI:** 10.1186/1742-6405-7-10

**Published:** 2010-04-23

**Authors:** Gita Ramjee, Nicola Coumi, Nozizwe Dladla-Qwabe, Shay Ganesh, Sharika Gappoo, Roshini Govinden, Vijayanand Guddera, Rashika Maharaj, Jothi Moodley, Neetha Morar, Sarita Naidoo, Thesla Palanee

**Affiliations:** 1 HIV Prevention Research Unit, South African Medical Research Council, 123 Jan Hofmeyr Road, Westville, 3630, Durban, South Africa

## Abstract

**Background:**

South Africa, with its scientific capacity, good infrastructure and high HIV incidence rates, is ideally positioned to conduct large-scale HIV prevention trials. The HIV Prevention Research Unit of the South African Medical Research Council conducted four phase III and one phase IIb trials of women-initiated HIV prevention options in KwaZulu-Natal between 2003 and 2009. A total of 7046 women participated, with HIV prevalence between 25% and 45% and HIV incidence ranging from 4.5-9.1% per year. Unfortunately none of the interventions tested had any impact on reducing the risk of HIV acquisition; however, extremely valuable experience was gained, lessons learned and capacity built, while the communities gained associated benefits.

**Experience:**

Our experience in conducting these trials ranged from setting up community partnerships to developing clinical research sites and dissemination of trial results. Community engagement included setting up community-based research sites with approval from both political and traditional leaders, and developing community advisory groups to assist with the research process. Community-wide education on HIV/sexually transmitted infection prevention, treatment and care was provided to over 90 000 individuals. Myths and misconceptions were addressed through methods such as anonymous suggestion boxes in clinic waiting areas and intensive education and counselling. Attempts were made to involve male partners to foster support and facilitate recruitment of women. Peer educator programmes were initiated to provide ongoing education and also to facilitate recruitment of women to the trials. Recruitment strategies such as door-to-door recruitment and community group meetings were initiated. Over 90% of women enrolled were retained.

Community benefits from the trial included education on HIV prevention, treatment and care and provision of ancillary care (such as Pap smears, reproductive health care and referral for chronic illnesses). Social benefits included training of home-based caregivers and sustainable ongoing HIV prevention education through peer educator programmes.

**Challenges:**

Several challenges were encountered, including manipulation by participants of their eligibility criteria in order to enroll in the trial. Women attempted to co-enroll in multiple trials to benefit from financial reimbursements and individualised care. The trials became ethically challenging when participants refused to take up referrals for care due to stigma, denial of their HIV status and inadequate health infrastructure. Lack of disclosure of HIV status to partners and family members was particularly challenging. Some of the ethical dilemmas put to the test our responsibility as researchers and our obligation to provide health care to research participants.

**Conclusion:**

Conducting these five trials in a period of six years provided us with invaluable insights into trial implementation, community participation, recruitment and retention, provision of care and dissemination of trial results. The critical mass of scientists trained as clinical trialists will continue to address the relentless HIV epidemic in our setting and ensure our commitment to finding a biomedical HIV prevention option for women in the future.

## Introduction

In 2007, Africa accounted for 67% of all people living with HIV worldwide, with 38% of all new infections occurring in the southern African region. The predominant route of HIV transmission in this region is reported to be heterosexual sex [1].

South Africa has the highest adult HIV prevalence worldwide - 17% in 2008 [2] - and an incidence rate of 1.4% per year (2005 figure) [3]. Some 33% of South African women aged between 25 and 29 years are infected with HIV, while KwaZulu-Natal is the province with the highest HIV prevalence among persons aged 15-49 years (26%) [2] and among pregnant women (39%) [4]. The high HIV prevalence and incidence rates coupled with good infrastructure make South Africa a location of choice in which to conduct HIV prevention intervention trials.

To date, several trials of HIV prevention technologies have been undertaken in the country, including those of male circumcision for HIV prevention [5], vaccines [6], microbicides [7-9], prevention of mother-to-child transmission [10,11], the vaginal diaphragm [12], and the Stepping Stone behavioural intervention trial [13], among others.

The HIV Prevention Research Unit (HPRU) of the South African Medical Research Council (MRC) has had the privilege of working with multiple sponsors to conduct four phase III and one phase IIb trials of HIV prevention technologies among women between 2003 and 2009 (Table 1).

**Table 1:** HIV prevalence and incidence of women participating in trials in KwaZulu-Natal, and overall trial outcome.

Trial (total enrolment)	Years	Location of HPRU sites (no. enrolled)	HIV prevalence (%)	HIV incidence (/100 woman years)	Overall outcome of trial: HR (95% CI)
MIRA (5045)	2003-2006	Durban (1515)	39	6.8	1.05 (0.84-1.32) P = 0.65

Carraguard (6202)	2004-2007	Durban (1485)	43	6	0.87 (0.69-1.09) P = 0.302

Cellulose sulphate (1428)	2006-2007	Durban (606)	51	5.29	1.61 (0.86-3.01) P = 0.13

HPTN 035 (3101)	2005-2008	Durban (702) Hlabisa (346)	21.6 28.2	4.6 9.1	0.71 (0.46-1.11) P = 0.1331

MDP 301 (9385)	2005-2009	Durban (2392)	36	6.1	1.05 (0.82-1.34) P = 0.712

HR = Hazard ratio

Methods for Improving Reproductive Health in Africa (MIRA) was a phase III trial assessing effectiveness of the latex vaginal diaphragm in prevention of HIV at three sites in southern Africa (two in South Africa and one in Zimbabwe) between 2003 and 2006 [12]. Final results suggested that the diaphragm had no effect on male to female transmission of HIV infection - hazard ratio (HR): 1.05 (95% confidence interval (CI) 0.84-1.32, P = 0.65) [12].

Between 2004 and 2007 the phase III trial testing Carraguard™, a lead microbicide product of the Population Council (New York), was conducted at three sites in South Africa: Isipingo in Durban, Soshanguve in Pretoria and Gugulethu in Cape Town. The results suggested that Carraguard™ had no effect on prevention of male to female transmission of HIV - HR: 0.87 (95% CI 0.69-1.09, P = 0.302) [8].

The phase III trial testing the effectiveness of cellulose sulphate (CS) in prevention of HIV among women at high risk was conducted at several sites in Africa (South Africa, Uganda and Benin) and a site in India between 2006 and 2007. The trial was stopped prematurely due to safety concerns during the first Data and Safety Monitoring Committee (DSMC) review in early 2007. Final results suggested that CS was not suitable for HIV prevention, with no statistically significant effect on safety - HR: 1.61 (95% CI 0.86-3.01, P = 0.13) [9].

Early in 2009 we heard of the positive but not significant effect of 0.5% PRO 2000 in the prevention of HIV transmission from males to females - HR: 0.71 (95% CI 0.46-1.11, P = 0.1331). The HPTN 035 study, sponsored by the Division of AIDS (National Institutes of Health), took place between 2005 and 2008 at two sites in South Africa (Durban and rural Hlabisa) as well as sites in Malawi, the United States of America, Zambia and Zimbabwe. The phase IIb clinical trial tested the effectiveness of two candidate microbicides: PRO 2000 (0.5%) and BufferGel against a placebo and a "no gel" arm [7].

The results of the multi-centre Microbicides Development Programme's (MDP) 301 trial [14] were announced in December 2009. The study tested 0.5% and 2% PRO 2000 against placebo. Unfortunately, PRO 2000, although safe, was found to have no effect on acquisition of HIV in this very large trial - HR: 1.05 (95% CI 0.82-1.34, P = 0.712).

The HPRU enrolled a total of 7046 women into these trials (Table 1), and due to the high HIV prevalence almost twice that number were screened prior to enrolment. There were a total of 561 HIV seroconversions among these women.

We report here our experiences in conducting these community-based HIV prevention trials in KwaZulu-Natal, South Africa, with a focus on strategies developed to ensure good data quality, completion of the trial within an ethical framework, and partnerships developed with participants, the broader community and other health care providers. Experiences with these trials have afforded us the opportunity to evolve methods for trial implementation which are subject- and location-specific, but which could also be adapted for use in implementing clinical trials elsewhere.

All of the trials were approved by local, national and international ethical and regulatory bodies.

## Community Engagement and Partnerships

### Setting up community-based research sites

The HPRU set up clinical research sites (CRS) in several communities in the greater Durban area and one site in a rural area; we aimed to conduct only one HIV prevention trial in any given area. Consultation and information sessions were held with community stakeholders and political and traditional leaders as part of the community-entry process, prior to development of the CRS. The community leaders in turn discussed the proposal with the respective community committees before approval was granted. Well before initiation or implementation of the study, community meetings were held to discuss aims, objectives and potential outcomes, and to provide the community with background education on HIV/AIDS prevention, treatment and care, study designs, ethics and regulatory processes. Community approval was sought in parallel with trial regulatory approval processes.

### Community profile and situational analysis of health services

Researchers' understanding of the community was enhanced by conducting a situational analysis of the demographics of the community members and the available health and education resources. The ethical obligation of clinical trialists to trial participants extends to ensuring that participants are able to access care for conditions which are beyond the purview or mandate of a research site. Thus the situational analysis included development of a referral log which documented the health and psychosocial care facilities available in the area surrounding the study sites. This included public and private care facilities, where researchers engaged facility managers to discuss the study and the potential need to refer participants for reproductive and other care. We were able to use this information to educate the community about the type of care that was available in their area, and to assist participants in accessing alternative facilities, should they be concerned about confidentiality issues arising from attending a facility in their own community.

### Education

Ongoing community education was provided at all levels, including traditional leaders, non-governmental organisations (NGOs), community-based organisations (CBOs), health care providers, women's groups, women and men.

We received requests from community members to provide education sessions on topics such as the diagnosis, treatment and management of tuberculosis (TB), high blood pressure and diabetes, as well as HIV/AIDS and other sexually transmitted infections (STIs). These education sessions were conducted at locations chosen by the community and were facilitated by the research clinicians, nurses, community liaison officers (CLOs) and outreach workers in the local language or English as required.

### Community Working Groups or Community Advisory Boards

Key stakeholders were identified to become part of Community Working Groups (CWGs) or Community Advisory Boards (CABs). Members of the CWGs were either volunteers or selected by the community, and were expected to represent the voice and interests of the community throughout the research process. CWG membership consisted of men and women between the ages of 30 and 60 years. Initially, CWG membership was dominated by men who held senior positions in the community - this has changed over time as more and more women became involved and empowered; most CWGs are now chaired by a woman. Women members have included teachers, managers of health care facilities, representatives from social development organizations, NGOs and CBOs. Male members have included political, traditional and religious leaders, as well as school principals. In total there were 20 members with equal gender distribution in each CWG. The education level of members varied from completion of high school to tertiary education.

In order to facilitate effective participation by CWG members, training was provided on the principles of ethics, human subject protection, informed consent material and process, and the study protocol. The CLOs employed by the HPRU organised regular monthly CWG meetings. CWG membership was voluntary but members were reimbursed for transport costs. A budget was allocated for CWG members to attend conferences, workshops and meetings both nationally and internationally, as part of the partnership with and capacity development of community members. We found that this level of capacity development was an incentive that assisted in retention of CWG members, who also provided feedback to the community on the events.

### Ongoing communication with trial site community

In addition to attending occasional CWG meetings, the Principal Investigator also provided feedback on the study progress at least twice per year, while key staff met with the community on an ongoing basis. Researchers and the CWGs were able to discuss challenges and concerns related to the HIV prevention research. Initially, community members (especially in the rural district of Hlabisa) expected researchers to provide infrastructure (roads, sanitation) and employment, and to redress poverty in the area. The role and contribution of researchers was clarified, emphasising the aim of the research protocol, and the importance of HIV prevention education and research to find safe, effective biomedical technologies to prevent HIV infection. Once this was clarified, no further requests were made.

Investigators held ongoing workshops, training sessions and meetings with CWG members and stakeholders throughout the duration of each of the trials (approximately 2-3 years). Clinical site staff also participated in community events such as Women's Day, World AIDS Day, and HIV awareness days.

### Myths and misconceptions

Since we set up CRS in 'research naïve' communities, there were many recurrent myths and misconceptions surrounding clinical trials, such as: "researchers collect blood to sell", "researchers infect women with HIV", "women are being used as guinea-pigs", "researchers pay the women to use the trial products", "[the researchers] put people and their families in danger using the GPS [Global Positioning System] machines", "government condoms have HIV", "the gel tightens the vagina", "trial staff encourage promiscuity among women" and "[colposcopic] images are placed on the Internet". Some of these rumours were published in local and community newspapers.

The most common myths and misconceptions were those involving transmission of HIV through research, misunderstandings concerning regulatory approval of clinical trials, and the selling of blood. Sporadic issues of this nature were encountered at all trial sites. We addressed these through outreach and education sessions at various levels. Participants received group education from CLOs, counsellors and nurses in clinic waiting rooms. Study staff were collectively trained on clear communication strategies. We also communicated with CWGs regarding myths and misconceptions as a means of conveying correct messages to a broader community. We attempted to provide additional HIV/AIDS education to the public during recruitment drives. Re-iteration of messages at various points assisted us in addressing these issues.

### Community participation in trial results messaging and dissemination

The ongoing relationships with community stakeholders and CWGs were extremely useful in formulation of a suitable language lexicon, messaging, and dissemination of trial results. Several months prior to the release of results, we initiated intensive education and information-sharing sessions to inform the community of trial completion and the possible outcome scenarios. The HPRU developed this dissemination plan following the reactions to closure of the CS trial [15]. Preparing the community well in advance and providing them with the possible trial outcome scenarios assisted us in discussing the final results with minimum challenges.

### Involvement of male partners

Although we were conducting trials of women-initiated HIV prevention options, we soon learned from the women that covert use was not culturally acceptable, especially in stable relationships. A strategy was used to engage men and educate them about clinical trials, HIV, STIs, safe sex and condom use. Several social events (such as soccer matches) were arranged at trial sites, after which education sessions were held to inform men of the research we were conducting. Involving men in the research process provided an opportunity for researchers to reduce the community's misconceptions about HIV prevention research. Male involvement also resulted in an increase in women volunteering to participate in the trial, increased product adherence, and facilitated communication in participants' personal relationships on issues specific to their sexual health. A few male partners also volunteered to come to site for HIV testing and counselling.

### Couple/partner workshops

We held several successful and interactive couples' workshops at the sites for study participants and their partners. Study clinicians, nurses, pharmacists and counsellors educated the couples on the trial protocol, HIV, STIs, condom usage, family planning, product adherence, and communication. The men were also offered voluntary counselling and testing (VCT) services, blood pressure/blood sugar testing, and measurement of body mass index.

## Recruitment

As described above, ongoing community education and outreach as well as networking with community stakeholders occurred before recruitment of women to the trials - which facilitated the recruitment process. We developed several recruitment strategies to ensure that we met our target accrual in the protocol-specified time. Strategies included having regular meetings with the community, targeting women's groups, getting approvals from service providers to recruit from family planning clinics, recruitment at Grant and Home Affairs offices (governmental agencies), and door-to-door and street recruitment.

During the recruitment process, field workers distributed ethics-approved participant information sheets. These included details of the study, such as study eligibility criteria, study procedures and laboratory tests, and information on the investigational products, such as the safety profile of the intervention. Study staff contact details were provided so that potential participants could access further information. In addition, community flyers in the local languages containing similar information were distributed throughout the recruitment areas. Senior staff contacted the heads of organisations such as health clinics, women's groups, CBOs and faith-based organisations to seek permission for field staff to recruit at these venues. Recruitment drives were also undertaken at large community meetings and fora such as World AIDS Day, World TB Day, Anti-Tobacco Day, etc.

### Peer education programme to assist with community education and recruitment

Trial participants who displayed a thorough grasp of trial procedures and who attended their scheduled trial visits were invited to join our Peer Educators programme, which was developed in 2005 with the approval of the local ethics committee. Their role was to provide education on HIV prevention, treatment and support, and to share their own personal experiences of trial participation. In addition, they undertook the responsibility of educating women within their residential area about the trial in order to facilitate recruitment. A total of 50 Peer Educators were trained, with the objective that these women would empower others on HIV prevention at community level. The Peer Educators not only assisted with recruitment and retention but also with formulation of trial results messaging and dissemination.

### The informed consent process

Informed consent (IC) is an ongoing process in clinical trials. In the microbicide trials conducted by the HPRU, both women and the wider community were provided with information and education on the study through the media of community awareness flyers, participant study information sheets, examples of the IC form, and at frequent question-and-answer sessions (conducted both at the clinic and at community meetings). As outlined by Woodsong *et al *[16], this "expanded model of informed consent" involves the community at the pre-enrolment stage, and subsequently the individual participant (at administration of the IC form), followed by continuous assessment of individual and community perceptions and reactions to the ongoing study.

We encouraged women to take an unsigned copy of the IC form home with them, so that they could discuss their possible participation in the study with their partners or families prior to enrolment. Once enrolled, a copy of the signed IC form was provided to participants for reference purposes, although they were advised that they could leave their copy at their clinic if they feared that it might be discovered by family members or partners to whom they had not disclosed their participation.

Assessment of understanding of the IC process has shown that the majority of women understood it. However, some women reported that they had initially experienced difficulty understanding medical or biological terms such as "gonorrhoea" or "syphilis", but were assisted by staff members [17]. There has previously been some indication that women might be experiencing a therapeutic misconception in relation to HIV prevention trials [18,19], and we endeavoured to continuously reinforce and assess comprehension of the blinded nature and objectives of the trials, and the role and contribution of participants.

The process of community engagement, including community awareness activities, engagement of local community leaders and appointment of the CWG, contributed to acceptance of the IC process instituted in our trials. We believe that this engagement has indicated respect for the local communitarian culture, and blended the more traditional Western and African concepts of selfhood to produce a form of IC which respects both the individual and the community [20]. However, we are aware that the community may still have concerns in this area - questions regarding the role of male heads of households in relation to consent of women family members to participate are among the most frequently asked at community feedback activities. We aim to continue to engage with the community to enhance the IC process, while also improving awareness of women's right to autonomy.

## Retention of Trial Participants

Retention of trial participants is crucial to ensuring that we have adequate statistical power to show an effect of the intervention. Our sponsors developed databases which sent reminders of missed visits to staff members. This information was communicated to field staff, who contacted women either telephonically and/or through home visits. In some trials we introduced an award system, which provided a gift equivalent in value to ~$2 for every six months of visits completed, or an award at the final visit for completion of the trial. In others we presented women with certificates of completion at their final visits. This level of acknowledgement was highly appreciated by study participants.

### Addressing participants' concerns to improve retention

Suggestion boxes were placed at each of the sites to allow women to voice their concerns anonymously, and suggestions were reviewed weekly by the site CLO. Some concerns raised by participants included long waiting times, unhappiness with performance of certain clinical procedures, discomfort at being examined by male doctors, etc. Participants were also encouraged to use a toll-free HPRU clinic number to reschedule their follow-up visits and/or report any problems which they may have experienced during participation.

### End-of-trial retention strategies

During the final stages of the trial, weekend clinics were held so that participants who were employed could be accommodated. Women who had relocated to other areas were transported to the clinic sites or to one of the remaining six HPRU trial sites for completion of visits.

To ensure high retention at the end of the trial, we conducted some close-out procedures "off-site" (with the woman's permission) at the woman's home, her place of work, or any suitable venue indicated by her [21]. Staff also travelled to other regions of South Africa to trace women who were enrolled in the trial. Location of women's residences was included in a geographic information system database (with their permission). This allowed us to identify women's homes and assisted in tracking those who had missed visits or were lost to follow-up.

Our strategies were successful in retaining over 90% of women in all our trials (Table 2).

**Table 2:** High retention rates were evidenced across the trials in KwaZulu-Natal.

Trial	Retention
MIRA (average at two sites)	94%
Carraguard	97%
CS	92%
HPTN 035 (average at two sites)	95%
MDP 301 (average at three sites)	96%

## Counselling

Counselling and testing is a basic component of screening, enrolment and follow-up for participants in clinical trials. Counselling, as a dedicated profession, is still a relatively new concept in sub-Saharan Africa. However, given the socially unstable South African context [22], the importance of counselling messages is vital. Counselling issues related to confidentiality, condoms, orphans, families, cultural beliefs and knowledge are of critical importance and need to be addressed. Consequently the role of counselling is paramount and given great consideration when conducting research.

In our clinical trial setting, three major types of counselling have been conducted: a) HIV and risk reduction counselling; b) contraception counselling; and c) study product adherence counselling. It was very important that the counsellor's approach towards participants was non-judgmental and conducted in a client-centred manner, and that the messages and approach evolved in response to contextual cues derived from confidential data on individual participants' experiences and circumstances. Counsellors constructed individual risk reduction plans through which each participant's progress was tracked and her future counselling needs profiled.

### HIV and risk reduction counselling

We aimed to use HIV testing as a means to promote behaviour change, in addition to its use as an eligibility tool during screening and enrolment. HIV education and pre-test counselling was achieved by providing information on: the difference between HIV and AIDS; modes of HIV transmission; methods of prevention; suggested frequency of testing in relation to an assessment of behavioural risk; the window period and its impact on test results; correction of myths and misconceptions; and verifying readiness for testing.

Similarly, risk reduction counselling was also client-centred: open-ended questions were asked, and counsellors followed techniques of active listening and probing to identify risk factors and barriers to risk reduction. HIV post-test counselling included provision and explanation of test results, explanations of additional testing which might be required as per the study protocol, and an assessment of the participant's understanding of the results.

### Contraceptive counselling

Contraceptive counselling on available interventions was implemented to assist the participant in choosing a method that she was most likely to adhere to. Nurses and counsellors were instructed to ask probing questions at screening or enrolment concerning the participant's pregnancy intentions and willingness to use a hormonal or barrier contraceptive method. Nurses were capacity developed and certified as providers of family planning at trial sites. This ensured availability of service provision at the trial site, which reduced the need for off-site referral for contraceptive uptake post-counselling.

### Adherence counselling

Study product adherence counselling commenced on the first day of enrolment into a particular trial. Counsellors and pharmacists provided standard information on the method of administration, mechanism of action, level of effectiveness, and advantages and disadvantages in the context of daily use of the study product concerned. Emphasis was placed on the blinded nature of the trial, and the necessity to practice safe sex with the use of condoms.

Follow-up counselling sessions were structured to assess adherence since the last session, provide appropriate levels of adherence counselling, develop strategies for the next month, and reinforce key messages. Given that adherence to product use is a major challenge in biomedical prevention trials, we suggest that it would be useful to assess the level of likely adherence to product use prior to enrolment by asking questions about, for example, adherence to contraceptives or STI medication as a proxy to assess potential adherence to new interventions.

## Community Benefits from Trials

Community benefits from a trial can be quantitatively determined at the end of the trial. There are several areas where the research participants (and therefore the community) benefit by having a trial conducted in their setting.

### HIV prevention, treatment and care education

We have provided HIV prevention, treatment and care education to approximately 90000 individuals across the seven communities where the CRSs are based. In addition, ongoing education continues to be provided as we implement new research studies at these sites. Peer education continues to be included in community activities on HIV/AIDS prevention.

### Ancillary care

Table 3 shows the approximate number of tests and procedures performed during each of the clinical trials undertaken. In the public sector in South Africa Pap smears are only offered to women over the age of 35, with three pap smears offered per woman. We offered approximately 11 000 Pap smears to women aged between 18 and 50 years. Prevalence of abnormal results varied from 10 - 20% across studies [23,24]. Anaemia was routinely identified in approximately 10% of the study population at baseline, while chronic active diseases such as hypertension and diabetes mellitus constituted <5% of findings. All participants were referred to our health care partners in the communities for care.

**Table 3:** Approximate number of tests and services provided to communities by HPRU during the trials.

Test	N	MIRA	Carraguard	CS	HPTN 035	MDP 301
Pap smear	11 624	1515	5609	-	2108	2392
STIs	136 623	44 572	39 723	16 585	9486	26 257
Pregnancy	94 594	14 857	17 133	8049	21 156	33 399
Colposcopy	299	-	-	-	299	-
Contraception*	2662	81 (5%)	678 (46%)	98 (16%)	872 (83%)	933 (67%)
CD4 count	120	35	-	-	-	85
**Total number of tests/services**	**245 922**	**61 060**	**63 143**	**24 732**	**33 921**	**63 066**

* Percentages are of participants in that trial on contraception.

In addition, some 136 000 STI tests were conducted (Table 3), average prevalence of STIs such as *Chlamydia trachomatis*, syphilis and *Neisseria gonorrhoeae *being 9%, 2% and 2% respectively at screening. A small percentage (c. 5%) of participants' male partners took up the offer of STI testing at the CRS. All women were provided with partner contact cards in an attempt to ensure that their partners also sought treatment for STIs.

HIV prevention trials target young women of reproductive age due to the high HIV incidence rates in this age group; unintended pregnancy in trial participants is a natural consequence. As per protocol, product use has to be suspended once pregnancy is detected, and this impacts as a loss of statistical power to determine product effectiveness. In order to retain women on product and avoid pregnancy, HPRU initiated provision of oral and injectable hormonal contraception at clinic sites. The options provided were aligned to those available from the local Department of Health, to ensure continuity of access post-trial. Uptake of contraception from HPRU sites varied widely across the different protocols (see Table 3).

HPRU staff counselled 2662 women who were not using any family planning methods at baseline and provided them with a contraceptive method of their choice. We found that many women were not aware of the range of contraceptive options available, and were therefore reluctant to go to family planning clinics.

### Social benefits

At Hlabisa we trained 150 home-based care-givers to assist communities with HIV-positive members who were chronically ill, and in Umkomaas we were successful in motivating for the opening of a municipal VCT centre. In addition, HPRU sourced funding to capacitate HIV clinics with counsellors and doctors in order to manage the burden of HIV in the community. The training provided to community members in HIV prevention research enabled them to seek jobs within the health sector in their community.

## Challenges Encountered

We faced a range of challenges in implementing these trials, and describe some of them below.

### Recruitment

As the community became more familiar with the study inclusion and exclusion criteria, non-eligible women began to manipulate their responses to facilitate enrolment during the screening visits. This included instances in which false declarations were made regarding use of contraception, intention to conceive, number of sex acts per month, previous instances of HIV testing (women were attending screening to confirm their HIV status), and dilution and swapping of urine samples collected for pregnancy testing (pregnant women swapped their samples with non-pregnant friends').

We believe this desire to participate in clinical trials was partially attributable to the high reimbursement rate of R150 for each scheduled clinic visit throughout the duration of the trial. This value has been predefined by the drug regulatory body, the Medicines Control Council (MCC) of South Africa. For women who have no other means of income, this may be the motivating factor encouraging enrolment into a study. Other reasons included a desire for personalised care and to avoid the long queues in the public sector centres/services. Despite this, we also believe that many participants were genuinely altruistic in their decision to join trials.

### Co-enrolment in other prevention trials

Given the high reimbursement rates (R150 per visit) approved by the MCC of South Africa, it was inevitable that participants would try to enrol in more than one trial, especially in a climate of high unemployment and disempowerment of women in this very patriarchal society. A confidential database was developed based on women's South African identity numbers, with a real-time check by staff on every woman screened (14 000) and enrolled in our trials (7046) [25]. Unfortunately, while we were able to control for co-enrolments at HPRU clinical trial sites through our internal checking system, we were not able to control for women enrolled in trials with other organisations.

In 2008 we identified 192 co-enrolments with another trial outside the HPRU. When women were questioned on their reasons for co-enrolling, they cited "financial incentives", "better care", and "wanting to register in another trial before ending on the current one". Fortunately, this did not impact on study outcomes. We hope to alleviate this problem in the future at trial sites in KwaZulu-Natal by instituting an electronic biometrics/fingerprinting system which will identify in real time any potential co-enrolments across sites and institutions. We will also continue to use our current system to monitor any co-enrolments at HPRU sites in addition to the fingerprint technology.

### Contraception use

Challenges to contraception uptake included participant apathy based on their own perceptions and beliefs, reluctance to take it due to lack of partner/family support, difficulty in adhering to the contraception schedules, and reluctance due to perceived side-effects such as weight gain. Contraception failure often occurred due to participants defaulting on contraception visits/schedules, and frequent method changes due to side-effects such as intermenstrual bleeding, nausea and weight gain. Some participants did not return to the clinics if they became pregnant, even though it was stressed that they could remain on the trial.

### Retention

A particular retention challenge was experienced in the rural area of Hlabisa where the population was highly mobile, and participants frequently migrated or relocated to urban areas in the province in search of employment. This necessitated follow-up attempts to locate the participants in urban areas, which were not always successful. Despite this, we were able to retain 96% of women in Hlabisa.

### Continuity of clinical care

#### Memoranda of Understanding with service providers to ensure continuum of care

A significant challenge during clinical trial implementation has been ensuring continuity of care of trial participants. During study participation, clinical management is governed by protocol-specified clinical procedures. Any aberrations from normal have warranted referral to local Department of Health clinics/hospitals for further clinical management.

The HPRU established Memoranda of Understanding (MOU) with the Provincial Department of Health and local/district health care providers under the jurisdiction of the overarching Provincial Department of Health MOU. The MOU ensures continuity of care of research participants for HIV and reproductive health care encompassing, among others, management of abnormal cervical cytology, management of intermenstrual bleeding, as well as management of incomplete and therapeutic abortions. The MOU also delineates referral for management and further investigations of variations from normal values of blood chemistries together with liver/renal and haematological readings.

### Inadequate health infrastructure

We visited our MOU partners on a quarterly basis to update them on the study. We were repeatedly informed about the lack of human resources to manage the high burden of HIV in the hospital and clinic settings. Managers at the referral system requested assistance with the employment of counsellors, clinicians, laboratory staff (especially to perform PCR for infants), and infrastructure development. We were able to provide some assistance but not all as requested due to a lack of appropriate mechanisms to ensure that specific health providers received the relevant funds.

Perhaps the way forward in bridging the divide between care provided in research settings and referral to Provincial Department of Health clinics is to enhance more health services buy-in to the research, by encouraging more active stakeholder involvement and modifying protocols to provide more local contextual relevance. In addition, an overlap or merging of research centres and local health care providers may prove to be a beneficial symbiotic relationship, ensuring research objectives are realised with relevance to the local health care context. This symbiotic relationship will bridge the divide between research and public health.

### Stigma and reluctance to seek care

Stigma and reluctance to seek care remain major problems at all trial sites. Participants who were HIV-positive at the outset were reluctant to seek care at their local clinics/hospitals for fear of friends and relatives finding out their HIV status, or due to denial of their test results. These fears were not without foundation; we have received reports from participants that clinic/hospital staff divulge confidential information to friends and family in the community. Furthermore, those that went to public clinics had to wait in long queues and join a specific HIV care programme which assessed disclosure, adherence to treatment and follow-up. Many participants believed that they were healthy and did not need to join the programme.

Reluctance to seek care was also associated with non-HIV clinical abnormalities which required clinical intervention at hospitals. Referral and/or appointments were made for further investigations and care. However, many women were not forthcoming with information about hospital visits, and upon enquiring at the hospital it was often found that referred participants had not actually attended.

### Care for HIV seroconverters

The HPRU provided care to participants who seroconverted while in the study for the duration of the trial. At the end of the trial, we were able to perform CD4 cell counts and viral load assays for some participants to assist with registering in the local HIV care programme (see Table 3). However, there was still reluctance to seek care, even though a detailed referral letter was provided. For example, we invited seroconverters enrolled in one of our trials to take part in a care programme which provided additional counselling and CD4 counts; of the 154 seroconverters, we found that 26% had already accessed public health care, 23% had enrolled in other treatment trials, 18% did not wish to participate, and 20% were not contactable [26].

We noted several instances of rapid HIV progression; the decision was taken to source private care since the long waiting lists in local clinics might mean these participants would only be attended to several months later. Furthermore, many women were reluctant to disclose their results to anyone, which meant that providing care became very difficult. These women relied more heavily on the trial staff and this raised concerns of dependency, especially at the conclusion of our trials.

The reason for HIV status non-disclosure was often found to be non-acceptance of an HIV-positive test result. Counsellors would engage participants to discuss their risk factors (e.g. unprotected sex, multiple partners, having a male partner with known multiple partners) and encourage the participants to come to the realisation that their risky behaviour (or that of their partners) had put them at an increased risk of acquiring HIV.

The counsellors also provided strategies to assist with disclosure, and encouraged the participants to identify potential family members or friends to whom they could disclose, and from whom they would receive emotional support. Disclosure was encouraged at each counselling session in the hope that participants would eventually feel confident enough to disclose their status. Some participants were financially reliant on their partners and were afraid that they would be left destitute if their partner found out that they were HIV-positive. Counsellors discussed employment prospects for these women and encouraged them to develop their own skills/capacity to allow them to become less financially reliant on their partners.

Many women did begin to feel more empowered due to the patience and encouragement of clinic staff and the education they received about being HIV-positive, the care and treatment available to them, and information about where such care could be accessed.

### Ethical boundaries of care in clinical research

During the course of these trials, we identified many cases in which the ethical boundaries regarding our responsibility to trial participants were unclear.

Referral to ancillary care has been a point of ethical tension. For example, a woman who had been screened out of the trial presented to us with a CD4 count of 20 cells per mm^3^. She was so ill that we had to transport and admit her to the local hospital. Some women became suicidal after learning of their HIV serostatus and expressed a desire to be cared for by the research site rather than local HIV care centres. We provided care for an HIV-positive participant who had contracted TB and was later paralysed. We sourced hospital and later hospice care for her.

We have also encountered participants who were not receptive to counselling or advice: a woman who was HIV positive continuously defaulted on her contraception; she had an ectopic pregnancy and had to undergo a termination. The same participant became pregnant again despite counselling, and had a premature baby which died soon after delivery. The same woman defaulted from the HIV treatment programme several times.

Despite efforts made by us to be ethically responsible, we realised that the current status of the South African health system caused many participants to rely on us for care, which tested our ethical boundaries of responsibility.

## Capacity Development

The HPRU collaborations and partnerships with major international organisations facilitated the development of a critical mass of clinical trialists, many of whom joined the MRC with a Master of Science degree and no knowledge of clinical trial implementation. Currently these world-class clinical trialists are Principal Investigators, sub-investigators, and Investigators of Record in new trials. In addition, research team members who joined the Unit as research assistants and fieldworkers were developed into CLOs, project coordinators and project leaders. A total of 300 staff were employed in these trials at our peak, many of whom were able to obtain higher degrees (Honours, Master of Science, Master in Public Health, and PhD). In addition, they attended international and local meetings and presented at scientific conferences.

We have developed a Good Clinical Practice (GCP) training programme that includes South African GCP guidelines and is provided by senior staff who are certified GCP trainers. All drivers are International Air Transport Association certified to transport hazardous material. In addition, staff have received training through sponsors on study implementation, quality control and quality management. The HPRU is recognised for its excellent data quality and exceptional chart notes in participant files. We were able to deliver good quality data and a high retention rate for all five of the trials undertaken between 2003 and 2009.

## Dissemination of Results

Dissemination of results was without incident if the tested intervention was found to have had no significant negative effect on risk of HIV infection. However, in cases in which a more complicated research outcome occurred, negative media reporting was often encountered, which severely impacted on community trust in the research organisation. Extensive efforts to remediate the image of clinical trials had to be undertaken. We have previously reported on this issue [15]. We believe that we need to communicate to the community from the outset what the possible outcomes of clinical trials may be, and such scenarios should be presented prior to commencement of the trial (Ramjee *et al*., unpublished).

## Conclusion

The HIV prevention field has so far had little success with biomedical intervention strategies. However, success also needs to be acknowledged in the changes which trial participants initiate in their lives following education and counselling sessions at CRS. This may spread to the wider community through their social networks.

Hypotheses generated for investigation in human trials were based on observations and pre-clinical animal and laboratory studies. Based on unsuccessful trial outcomes, it is difficult when implementing new trials to convince the community that evidence for the new concept also comes from data generated from animal or laboratory studies.

Recent failures with interventions such as vaccines [6], microbicides [8,9,27], HSV2 suppressive therapy [28,29] and vaginal diaphragms [12] have been extremely difficult for the field as a whole. Unfortunately, with high HIV incidence rates among young women, decreasing the effort to find an effective HIV prevention intervention is not an option. The community and all our stakeholders need to understand these challenges and support continued research on HIV prevention.

We urgently need political will and commitment together with a co-ordinated effort in partnership with scientists, civil society, activists and other relevant organisations to address the unacceptably high HIV incidence rate in our setting. The lessons learned here provide invaluable insights into conducting clinical trials in middle-income countries such as South Africa. Our experience and lessons learned will be of importance to others in similar settings. With a critical mass of scientists developed as world-class clinical trialists, we hope to build on the lessons learned as we move forward to test new HIV prevention interventions for women and men.

## Competing interests

The authors declare that they have no competing interests.

## Authors' contributions

GR was the PI and is Director of the HPRU, and oversaw implementation of all trials and studies mentioned, as well as drafting of the manuscript; NC, ND-Q, Shay Ganesh, Sharika Gappoo, RG, RM, SN and TP contributed to the implementation of the trials and shared experiences, as well as data on participant retention and HIV prevalence and incidence. Shay Ganesh provided information on clinical challenges, while VG provided the content of the sections on counselling in the South African context. JM provided contraceptive data. NM provided information on community entry and interaction.

All authors have read and approved the final version of the manuscript.
